# Empowering Mental Health Management: A Participatory Approach for Black Communities Using Psychological Ownership

**DOI:** 10.1111/hex.70585

**Published:** 2026-02-10

**Authors:** Clarissa Gardner, Weston Baxter

**Affiliations:** ^1^ Dyson School of Design Engineering Imperial College London London UK; ^2^ Institute of Global Health Innovation Imperial College London London UK

**Keywords:** cultural sensitivity, empowerment framework, health equity, mental health disparities, participatory action research

## Abstract

**Introduction:**

Black British communities experience significant mental health disparities, driven by systemic racism, stigma and a lack of culturally competent care. Psychological ownership (PO) – the sense that something is ‘mine’ – offers a novel framework for understanding mental health empowerment and participatory care. This study examines PO as a lens for unpacking barriers and opportunities for empowerment and translating these insights into participatory design solutions.

**Methods:**

We conducted semi‐structured interviews and co‐design workshops with Black British mental health service users and staff at a culturally specific recovery service. Using PO theory, we analysed participants' motives (efficacy and effectance, self‐identity, and having a place to dwell) and routes (control, intimate knowledge, and self‐investment) for taking ownership of their mental health management.

**Results:**

Key findings confirmed the usefulness of framing understanding mental health empowerment from the perspective of psychological ownership and revealed the importance of culturally grounded spaces, peer support, and participatory decision‐making. Co‐design workshops further contextualised these findings, resulting in actionable intervention concepts.

**Conclusion:**

By integrating theoretical insights with participatory processes, this study highlights the potential of PO to guide the design of equitable mental health services. It concludes with recommendations for embedding PO principles into culturally competent healthcare models.

**Patient or Public Contribution:**

The study was conducted at a mental health day service in London, which exclusively serves people of African and Caribbean descent. There is a committee of service users who are involved in providing input and feedback on the delivery of services at the centre. This group was engaged throughout the study, from reviewing the study proposal and materials to engaging in the analysis of interviews and taking part in the codesign activity.

## 1 Introduction

Mental health disparities in the United Kingdom are pervasive, with Black African and Caribbean communities disproportionately affected by systemic inequities. While prevalence rates of psychotic illness are comparable across ethnic groups [1], Black individuals are up to four times more likely to be detained under the Mental Health Act 1983 and overrepresented in secure psychiatric facilities [2]. These disparities are compounded by barriers to accessing mental health services, including stigma, systemic racism, and cultural mismatches in care [3, 4, 5, 6, 7].

Black British communities often face a dual burden: systemic inequalities that affect access to quality care and cultural stigmas around mental health that deter individuals from seeking help [3, 4]. Mental health services frequently fail to address these complex realities, with care models that are either culturally inappropriate or underpinned by Western‐centric assumptions about recovery [1]. For example, research highlights that Black individuals report feeling alienated in mainstream mental health settings due to cultural insensitivity and a lack of representation among healthcare staff [1]. This results in poorer outcomes, higher relapse rates, and diminished trust in healthcare systems [3, 8, 9, 10].

Participatory models of care have gained traction as a way to empower marginalised groups by prioritising user engagement, self‐management, and shared decision‐making [11, 12, 13]. These models hold promise for bridging the gap between users and providers by ensuring that healthcare interventions are co‐created with input from the communities they aim to serve. Participatory design methods, in particular, offer practical approaches for involving service users in the development of culturally grounded mental health services [9, 10, 11, 14, 15]. However, there is a need for frameworks that systematically guide these efforts, particularly for Black British communities.

In health research and practice, empowerment definitions vary but can commonly relate to greater control over decisions and actions affecting their health, supported by appropriate knowledge and a supporting environment [16, 17]. Importantly, contemporary empowerment frameworks emphasise that empowerment is not achieved through the transfer of responsibility to the individual in isolation but through the alignment of individual agency and systems of care. For example, the EMPATHIE project identifies patient empowerment as a moment where patients have knowledge, take action and have control over their condition. The report also identifies strategies aimed at both patients and professionals [18]. Similar principles underpin recovery‐oriented and patient activation approaches in mental health [11, 12], which frame empowerment as relational and system‐enabled rather than purely individual. Psychological ownership (PO) is introduced here not as an alternative to these empowerment frameworks, but as a complementary lens that helps specify how empowerment is experienced and enacted in everyday mental health management.

Psychological ownership (PO) – the sense that something is ‘mine’ – may offer a robust framework for understanding and fostering empowerment in mental health management. Originating in organisational psychology, PO has been used to explore behaviours linked to control, responsibility, and attachment among others [19, 20]. It has been adapted for healthcare contexts to explain how individuals feel ownership over their health [21, 22], and for the co‐creation of general wellbeing [23], yet its application to mental health remains underexplored. The theory of PO identifies three motives for ownership: efficacy and effectance (the desire to control one's environment), self‐identity (the expression of oneself through ownership), and having a place to dwell (a sense of belonging) [20]. These motives are fulfilled through the routes of control, intimate knowledge, and self‐investment [20].

In healthcare, fostering psychological ownership has been linked to improved self‐management and engagement with treatment [21, 22, 24, 25]. However, few studies have applied PO to understand the unique challenges faced by marginalised populations, particularly in designing culturally competent care for Black communities. Research suggests that culturally grounded services can address these gaps by integrating culturally sensitive practices and fostering community support [1, 3].

This study investigates how PO can be used to understand and address barriers to mental health empowerment in Black British communities. Using interviews and co‐design workshops with service users and staff at a mental health day service exclusively for people of African and Caribbean descent, we explore how PO motives and routes manifest in participants' experiences and how they can inform culturally competent, participatory interventions. By integrating theoretical insights with participatory processes, this research bridges the gap between understanding ownership and designing actionable, equitable mental health solutions.

## 2 Materials and Methods

### 2.1 Study Design

This qualitative study employed a participatory action research (PAR) approach to explore the role of psychological ownership (PO) in fostering mental health empowerment among Black British communities. PAR was chosen for its ability to actively involve participants in identifying barriers to mental health management (MHM) and co‐developing culturally relevant solutions [26, 27]. The research consisted of two phases: semi‐structured interviews to understand participants' experiences with MHM, framed through the PO framework, and co‐design workshops to collaboratively translate interview findings into actionable intervention concepts.

### 2.2 Setting

The study was conducted at a mental health day service in London, herein referred to as The Centre. The Centre provides outreach through local general practitioner surgeries and mental health wards, and recovery support exclusively for people of African and Caribbean descent who identify as politically Black. The design of the physical space and the services that are delivered by The Centre address many of the systemic barriers that Black individuals face in mainstream mental health services [3]. The Centre provides culturally sensitive mental health recovery support, delivered by staff of African and Caribbean descent, including activities such as African drumming and hearing voices groups.

### 2.3 Participants

Participants included 12 service users with lived experience of mental health challenges and six staff members involved in service delivery at The Centre. Eligibility criteria for service users included self‐identification as Black African or Caribbean, being over 18 years of age, and current or recent engagement with The Centre's services. Staff members were selected for their insights into cultural and organisational barriers, and availability across the duration of the project. A purposive sampling strategy ensured diverse perspectives based on age, gender, duration of engagement with mental health services, and mental wellness at the time of the interview and workshop.

The study was also supported by the service user committee at The Centre, who were consulted from the inception of the study through to the delivery of the final service design concept, to support decision‐making and ensure the study was conducted aligned with the proposal.

### 2.4 Data Collection

The study employed two phases of data collection. In the first phase, semi‐structured interviews were conducted with all 18 participants to explore their experiences with MHM. The interview guide was informed by the PO framework, focusing on the motives for ownership (efficacy and effectance, self‐identity, and belonging) and the routes to ownership (control, intimate knowledge, and self‐investment). Example questions included: ‘To what extent do you feel you have control over the management of your mental health?’ ‘How does the ability to manage your own mental health make you feel as a member of your community or society in general?’ and ‘To what extent do you feel that others help you to manage your condition?’.

The second phase consisted of two co‐design workshops with seven participants, comprising service users selected from the interview cohort based on their interest in co‐design and their availability. Workshop activities included reviewing interview findings to confirm their relevance and completeness, brainstorming sessions to translate PO principles into culturally grounded intervention concepts, and the collaborative development of initial sketches and descriptions of proposed solutions. These workshops emphasised participatory decision‐making, ensuring that all voices were heard and valued.

### 2.5 Data Analysis

Data from both phases were analysed thematically using the PO framework. Interview transcripts were coded deductively using NVivo 12 (released 17, Lumivero) [28] based on PO dimensions, including motives such as efficacy and effectance, self‐identity, and belonging, as well as routes like control, intimate knowledge, and self‐investment. Additional themes outside the PO framework were coded inductively to capture emerging insights. Emergent themes were initially coded by the first author and discussed by both authors at regular intervals throughout the study. Results were further discussed and shared with the committee and participants who expressed interest in further discussion about the progression of the research.

Outputs from the workshops, such as concept sketches and group discussions, were analysed to identify how PO principles were operationalised in proposed interventions and to evaluate their cultural relevance, feasibility, and alignment with interview findings. To ensure consistency and depth, all data were triangulated, and initial coding was reviewed collaboratively by the research team.

### 2.6 Ethical Considerations

A lay summary of the study proposal, the participant information sheet and consent form, and interview questions were reviewed by the committee for their suggested amendments and approval. Ethics approval was then obtained from Imperial College Research EthicsCommittee (ICREC: 20IC6144).

Due to the relatively small size of the service and the small number of participants in this study, demographic data was not collected due to the increased risk of service users being identified.

The study was conducted while some restrictions related to the COVID‐19 pandemic were still in place, and concerns remained for individuals. In response to these national and individual requirements, the interviews were conducted remotely, and the co‐design participants were offered a remote or in‐person option.

The Centre staff played an instrumental role in safeguarding the well‐being of service users who were taking part in the interviews and supporting the facilitation of the in‐person codesign workshop.

Participants were informed about the study's objectives, their right to withdraw at any time, and the measures taken to ensure confidentiality. Co‐design activities were conducted with sensitivity to participants' mental health needs, including providing breaks and support as required.

Funding was retrospectively acquired from the Institute of Global Health Innovation, Imperial College London and used to pay service user participants compensation for taking part in the interviews and codesign sessions based on the National Institute for Health Research's payment guidance for researchers and professionals [29].

## 3 Results

The findings from this study are presented in two parts. First, insights from semi‐structured interviews are organised using the psychological ownership (PO) framework. Participants' experiences with mental health management (MHM) are discussed through the PO dimensions: motives (efficacy and effectance, self‐identity, and belonging) and routes (control, intimate knowledge, and self‐investment). Together, this presents a compelling understanding of why and how someone does(n't) manage their mental health as illustrated in Table 1. There are positive and negative statements in the table, which act as summaries of the themes emerging from the research. Second, the results of the co‐design workshops illustrate how these insights were translated into actionable intervention concepts, with a focus on cultural relevance and participatory approaches.

**Table 1:** A mental health management empowerment framework based on psychological ownership theory.

Psychological ownership theory	Interview findings
Target of ownership	Managing my mental health is MY responsibility
**Motives to own – why I (do not) want to own my mental health management**	
Efficacy and effectance (E + E)	I need to take an active role in my mental health management because no one else will be able to make as much of an impact I can cope with other aspects of my life if I take an active role in my mental health management I can minimise the likelihood of hospitalisation if I take an active role in my mental health management I want to effectively self‐manage my mental health so I can be a positive influence on others who may still be struggling (−) I feel like by seeking support from others in managing my mental health I will be perceived as a burden or stigmatised and I will lose my personal relationships (−) I do not feel prepared to be discharged from a mental health service because I do not think I can manage my mental health without their support (−) I am concerned that if I start taking medication for my mental health, I will be reliant on it for the rest of my life (−) I feel discouraged from speaking about my mental health problems or acknowledging my mental health needs
Self‐identity (S‐Id)	I see and accept myself as someone living with a mental health condition as opposed to a mentally ill person I see myself in the people who are trying to support me, and the possibilities for my mental health recovery I see myself as someone who could function and contribute to my community (−) I cannot see myself as someone who could recover from mental illness (−) I worry that my mental health challenges will lead others to associate me with negative stereotypes about people from my community (−) I do not want to follow through with treatment advice because it conflicts with my religious or cultural beliefs.
Having a place to dwell (HaptD)	I feel comfortable with my team of mental health professionals and peers because I can relate to them I have a support system who can recognise when I need support and provide suggestions of what I can do (−) I have no one close to me who knows I have a mental health condition or understands the importance of mental health management (−) I feel disheartened when I am discharged from a mental health service because I feel like I am losing access to support (−) I feel judged by mental health professionals and people in my community when I share my mental health challenges
**Routes to own – how I have ownership of my mental health management**	
Control (C)	I make decisions about how I want to manage my mental health
*Roles and responsibilities*	I influence how my mental health services support me I decide under what circumstances I need to enter hospital I choose who to disclose my mental health condition to I decide who can advocate for me if I am too unwell to look after my mental health I decide what role medication plays in my mental health management
Intimate Knowledge (IK) *Knowledge*	I know what my symptoms and triggers are I know when I can manage at home versus when I need to enter hospital I know what medication and dose works for me, and how my medication effects my mind and body I have expert knowledge of my diagnosis, symptoms and triggers
Self Investment (S‐In) *Actions and effort*	I actively combat isolation/build and maintain relationships I make myself look presentable I build a positive relationship with my identity as part of my mental health management I take my medication as prescribed
Ability to own	I have a general awareness of and knowledge about mental health and equate its importance to my physical health
	I have the mental capacity to make decisions about my mental health management and follow through with my self‐management techniques
	I have an open attitude towards seeking help and trying different self‐management strategies as part of my recovery journey
	I have the communication skills and confidence to express my needs and wants to my mental healthcare team and service providers
	I have a mindset where I can see it as a positive thing if I am discharged from a mental health facility because I am well enough to manage on my own
	I have a positive relationship with all aspects of my identity
Opportunity to own (attributes and features)	I need necessities in my life to remove the lifestyle factors that could be contributing to my mental health challenges (4 worlds)
	I need to know what service provisions and treatments are available for me to choose from.
	I need a mental healthcare team that believes in and uphold person‐centred care and recovery
	I need a mental health service provider or care team that understands my background and identity, and how this influences my experience of mental health services and my relationship with mental health management
	I need reliable resources to educate myself about my mental health condition(s) and treatment(s)
	I need activities that I can do to combat boredom and isolation.
	I need a support system who are recognised by the mental healthcare system as my advocates
	I need a mental healthcare team who will hold me accountable and uplift me to maintain good hygiene and presentation
	(−) Lack of societal/community awareness and education about mental health. Including the fear of danger associated with mental illness
	(−) Receiving the wrong diagnosis or being prescribed the wrong treatment
	(−) Decrease in funding for locally managed mental health services
	(−) High turnover of staff
	(−) Poor communication between services
	(−) Reduced community/in‐person mental health services as a result of the COVID‐19 pandemic restrictions

*Note:* (–) denotes the experiences and contributing factors that challenge or prevent people from feeling a sense of ownership over their mental health management.

This framework specifies the target of ownership, the motives that encourage or discourage ownership and the routes that are actual actions to take for ownership to occur. It also lists the capabilities required by an individual and the opportunities that need to be afforded to them.

### 3.1 Insights From Interviews: Empowered Mental Health Management

#### 3.1.1 Managing My Mental Health Is My Responsibility

Participants consistently identified managing their mental health as a personal responsibility that should be carried by the individual and supported by recovery support workers, clinical teams and the broader support network. There was acknowledgement that individuals with conditions that are considered severe such as bipolar disorder and personality disorders, face nuanced challenges in owning their mental health management, particularly if such individuals are not actively engaging with an appropriate service. However, interviewees agreed that it is important to let individuals decide their level of participation and for healthcare professionals and support networks to avoid adopting a paternalistic approach to care. The resulting target of ownership is therefore understood as the responsibility to manage one's mental health.

“I personally think I am 100% responsible for managing my mental health. The team around you play a significant role but it all starts and ends with you” – service user.

#### 3.1.2 Why I (Do Not) Want to Own My Mental Health Management

The psychological ownership framework provided a valuable lens for understanding why participants either took or did not take ownership of their mental health. By exploring the motives of efficacy and effectance, self‐identity, and having a place, the framework captured a wide range of reasons behind participants' behaviours and attitudes. This theoretical structure was particularly useful for organising and analysing the diverse and sometimes ambivalent experiences shared by participants, offering insights into both enablers and barriers to ownership.

Participants identified several reasons for wanting to take ownership of their mental health. Efficacy and effectance was manifest in the belief that their participation in managing their mental health has a greater impact on their recovery journey than anyone else's contributions. They found empowerment in tracking symptoms and recognising triggers, which allowed them to pre‐empt crises and feel in control. Staff also spoke of motivating members to take an active role in their mental health by illustrating that they could be a positive example to other Black people who are apprehensive about seeking mental health support. Self‐identity was also a key motivator, with participants describing how shifting their view from seeing themselves as a person living with a mental health condition as opposed to merely being a mentally ill person was part of what allowed them to see themselves as capable and accepting of themselves. Lastly, a sense of having a place to dwell provided a powerful incentive for ownership, with many participants valuing spaces like the The Centre for offering safety, support, and cultural understanding.

“Have someone to contact who is not your doctor (e.g., friend or neighbour). They might be able to talk reason. Maybe that's all you need and to know that people care.” – service user

Conversely, participants also highlighted reasons they felt unable or unwilling to take ownership. Participants felt disempowered during transitions where they felt less capable of managing without adequate support or they cited systemic and cultural barriers, such as stigma within the Black community, which framed mental health challenges as a weakness and discouraged open discussion. Some described self‐identity as a hindrance, noting how internalised stigma led to feelings of shame and denial about their condition. Others pointed to the absence of people they felt they could talk to about their condition as a threat to the belongingness captured in the having a place to dwell motive. This lack of representation and understanding left them feeling unsupported and, in some cases, disempowered.

“We provide an open platform to discuss the challenges of what it is to be a Black person engaging with the mental health system … when some members are unwell, they get stigmatised or pigeonholed and then that is internalised.” – staff member.

#### 3.1.3 How I Have Ownership of My Mental Health Management

The psychological ownership framework was instrumental in identifying the pathways through which participants developed or failed to develop a sense of ownership over their mental health. By focusing on the routes of control, intimate knowledge, and self‐investment, the framework provided a clear structure for understanding how participants' behaviours and experiences shaped their feelings of ownership. These routes revealed both actionable strategies for fostering ownership and significant barriers that needed to be addressed.

Participants described several ways in which they successfully developed ownership of their mental health. Control was a critical pathway, with participants emphasising the importance of autonomy in managing their care. Decisions such as whether to disclose their mental health condition, when to seek professional support, and how to approach treatment were seen as empowering. Intimate knowledge also played a key role, as understanding their symptoms, triggers, and coping mechanisms enabled participants to feel more in command of their mental health. Finally, self‐investment was highlighted as an essential route to ownership, with participants attributing their progress to the effort they put into activities like attending therapy, maintaining relationships, and adhering to treatment plans.

“A lot of our work involves getting them to admit themselves voluntarily rather than getting sectioned [under the Mental Health Act].” – staff member

However, participants also identified barriers that undermined these routes to ownership. Control was often constrained by systemic paternalism, where healthcare professionals made decisions without involving the individual. This left participants feeling disempowered and disengaged as the management of their condition was being done to them but not with them. Intimate knowledge was sometimes inaccessible due to a lack of culturally relevant resources, leaving participants to navigate their mental health on their own. Similarly, the ability to engage in self‐investment was hindered by external factors such as stigma, social isolation, and limited access to community‐based support systems. These barriers collectively undermined participants' ability to fully own their mental health.

“It took a long time for me to find the right medication that worked for me. I had an understanding psychiatrist who could see that I was trying to help myself – I think that is really important.” – service user.

#### 3.1.4 What Enables Me to Have Ownership of My Mental Health Management

The routes to psychological ownership—control, intimate knowledge, and self‐investment—highlighted the dynamic interaction between individuals and the environments in which they live. Ownership was not solely an individual process but was shaped by the opportunities and constraints afforded by participants' circumstances. For instance, the ability to exercise control often depended on how much autonomy healthcare systems granted, while the development of intimate knowledge was facilitated or hindered by access to culturally relevant resources. Similarly, participants' capacity for self‐investment was influenced by the support available within their social and community networks. This interplay underscored that ownership is co‐constructed, arising from both individual agency and the structural and social environments that shape recovery experiences. Findings relating to the abilities and opportunities needed to successfully manage one's mental health are outlined below.

The ability to take ownership of mental health was rooted in individual capacities, including an awareness of mental health conditions, mental capacity, and an open attitude toward addressing challenges. Participants described the importance of communication skills, which allowed them to articulate their needs to both healthcare providers and support networks. Confidence and a positive mindset were also essential, as these traits empowered individuals to engage actively with their recovery. A positive relationship with their identity, particularly in the context of being Black, was another significant factor. For many participants, this relationship fostered resilience and enabled them to reconcile cultural expectations with their personal experiences of mental health. When these abilities were present, participants felt better equipped to take responsibility for their mental health and navigate the complexities of recovery.

Opportunity to take ownership was shaped by the external environment, particularly the availability of resources and the structure of support systems. Participants emphasised the importance of knowing the available treatments and having access to a mental healthcare team that supported person‐centred care. Trust was enhanced when professionals understood participants' cultural backgrounds, which made them feel respected and validated. Reliable sources of education, particularly those tailored to their cultural context, were critical in fostering a deeper understanding of their mental health. Beyond formal care, participants highlighted the value of structured activities that prevented boredom or negative mental health triggers, as well as networks of support that held them accountable. For some, these opportunities created a foundation for ownership by bridging the gap between personal effort and systemic support. However, where these opportunities were lacking – due to inaccessible services, cultural insensitivity, or social isolation – participants often felt disempowered, highlighting the crucial interplay between ability and opportunity in fostering psychological ownership.

### 3.2 Towards a Participatory Approach to Culturally Relevant Mental Health Service Design

The co‐design workshops were pivotal in translating the findings from the interviews into actionable intervention concepts. Participants – including service users and staff from The Centre – were actively involved in validating insights and addressing systemic barriers that hinder mental health ownership in Black British communities. The workshops built upon the themes identified during the interviews, with a focus on fostering autonomy, belonging, and intimate knowledge in mental health management. During the workshops, participants confirmed the relevance of key themes identified in the interviews. Autonomy in mental health management was strongly reaffirmed, with participants emphasising the importance of having control over decisions related to their care.

To promote equity in the codesign process, the researcher conducted a series of knowledge exchange exercises around ideation, and assumed an administrative responsibility in gathering ideas and presenting them back to the group to support their decision‐making. Through collaborative discussions, several intervention concepts were co‐designed to align with the psychological ownership (PO) framework. Key ideas included My Mind Care Plan, a personalised self‐management tool designed to empower users to understand and manage their mental health more effectively, and Peer Support Service, a three‐point service which connects the user with a registered mental health advocate, a ‘buddy’ with lived experience of mental health recovery, and a family mediation programme. Another concept, Good Care Hub, is a social space for the Black Community focused on fostering cultural pride and signposting relevant mental health support, providing education to reduce stigma around mental health within the Black community.

**Figure 1:**
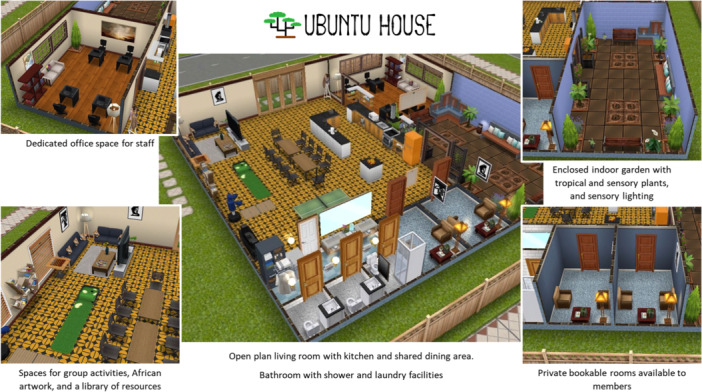
Screenshots of the Ubuntu House concept which was created using The Sims FreePlay^TM^. The physical space has been designed to prioritise the PO motive of having a place to dwell as a homely space for communal gatherings (an open plan kitchen with large dining area), respite (a sensory garden) and opportunities to relate to one another (an area for group activities and culturally specific artwork).

**Figure 2:**
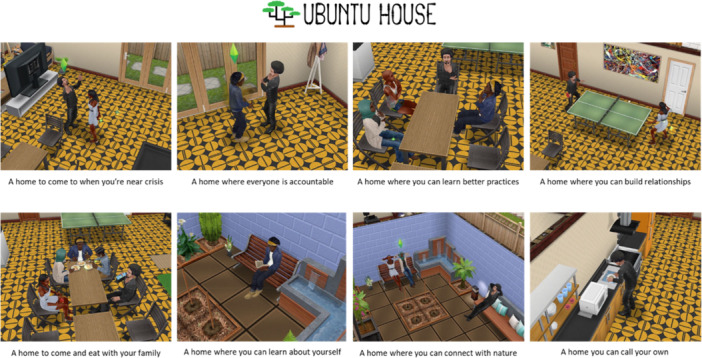
Screenshots of the intended interactions that would take place within the Ubuntu House concept, created using The Sims FreePlay^TM^, which relate to the PO routes to ownership of control (members have an option other than the hospital as a place to come when they are in crisis), intimate knowledge (members can access a library of culturally relevant mental health resources), and self‐investment (members can actively combat loneliness and build positive relationships with their community.

The standout concept, Ubuntu House, emerged as the most comprehensive intervention developed further with the participants. Ubuntu is a word derived from Nguni and Bantu languages and can be expressed as, ‘I am because of who we are.’ Ubuntu House was envisioned as a community‐oriented, non‐clinical space that prioritised the PO motive of belonging by fostering an environment of shared experiences and inclusivity. It was designed to offer peer‐led groups, culturally tailored programming, and activities aimed at reducing isolation and promoting empowerment. The Sims FreePlay^TM^ online game was used to illustrate the final concept to the participants [30] (Figures 1 and 2).

Beyond belonging, Ubuntu House addressed other PO dimensions. It supported control by providing opportunities for participants to co‐create their care plans and contribute to the design of its programmes. It fostered intimate knowledge through access to workshops and resources that encouraged participants to better understand their mental health conditions and strategies for recovery. Finally, Ubuntu House enabled self‐investment by encouraging active participation in its operations and programming, giving participants a tangible stake in their mental health management.

Participants also reflected on potential challenges and offered refinements to the proposed concepts. For Ubuntu House, sustainability emerged as a primary concern, particularly regarding long‐term funding and operational support. Suggestions included partnering with local organisations to share ownership and financial responsibility.

Overall, the co‐design workshops demonstrated the value of participatory methods in addressing systemic barriers and translating theoretical insights into culturally tailored solutions. By aligning each intervention with the PO framework, the workshops highlighted the potential for these concepts to empower individuals to take ownership of their mental health while addressing gaps in current mental health services. Ubuntu House, as the most developed concept, offers a promising model for culturally competent and inclusive mental health care, with further testing and refinement necessary to realise its full potential.

## 4 Discussion

This research explored how psychological ownership can enhance mental health management within Black communities, using participatory research methods and co‐design processes. Through the involvement of members and staff from The Centre, the project investigated systemic challenges, identified enablers of ownership, and designed a proof of concept, Ubuntu House, as a culturally tailored intervention. The findings contribute both theoretical and practical insights into fostering psychological ownership in mental health care.

### 4.1 Theoretical Implications

The findings of this project reinforce the utility of the psychological ownership framework for understanding mental health empowerment. The framework provided a robust lens for analysing why individuals take or do not take ownership of their mental health and highlighted how motives (efficacy and effectance, self‐identity, and belonging) and routes (control, intimate knowledge, and self‐investment) intersect with systemic barriers. Importantly, participants consistently expressed that recovery hinges on taking ownership, exemplified by the sentiment: *‘*Managing my mental health is MY responsibility.’ This underscores the framework's relevance to health behaviours, particularly within underserved populations.

Rather than positioning PO as a novel form of empowerment, this study demonstrates how it can function as an integrative framework that operationalises empowerment in practice. The antecedents outlined in Table 1 closely mirror those identified in established health empowerment models, including EMPATHIE, while extending them by explicitly accounting for identity and belonging. This extension is particularly important in mental health contexts shaped by stigma, cultural marginalisation and coercive care pathways. This, together with a range of developed measures of PO and a rich body of literature on PO theory, shows how PO moves empowerment towards useful ways to frame, evaluate and guide empowerment interventions.

Additionally, the framework's ability to capture the dynamic interaction between individuals and their environments deepens our understanding of ownership as co‐constructed. The findings demonstrated how external factors, such as culturally relevant spaces and supportive networks, shape the routes to ownership. This adds nuance to existing theoretical applications of psychological ownership, suggesting that it is not solely an individual construct but one that relies on environmental affordances. The project also highlights the role of identity in ownership, particularly for marginalised groups. For many participants, a positive relationship with their Black identity strengthened their sense of ownership, while cultural stigma often undermined it. This suggests that psychological ownership is intricately tied to social and cultural contexts, warranting further exploration in diverse populations.

### 4.2 Practical Implications

The participatory nature of this project provides important insights for designing culturally competent mental health interventions. Ubuntu House exemplifies how the psychological ownership framework can be operationalised to address gaps in care for Black communities. Drawing on The Centre's person‐centred approach, Ubuntu House integrates features such as culturally sensitive environments, peer‐led support, and activities that promote self‐management and belonging. These design principles align with best practices identified in voluntary sector mental health services for ethnic minority communities, such as user involvement, collaboration with local services, and the fostering of cultural identity throughout care.

Incorporating participatory design methods ensured that the intervention reflected the lived experiences of its intended users. Members and staff at The Centre played an active role in reviewing findings, co‐producing the design brief, and shaping the final concept. This level of involvement not only enhanced the cultural relevance of the intervention but also fostered trust and buy‐in among participants. One participant remarked: ‘You'd come here and ask questions: ‘Should it look like this? Should it look like that?’ then you'd go away, change it a bit, and come back to ask, ‘Is this right?’ That's how it should be done.' This feedback underscores the importance of co‐creation in designing solutions that resonate with underserved communities.

The practical insights from this project also extend to policymaking and funding [1, 26]. Programmes like Ubuntu House aligned with national initiatives at the time of conceptualisation, such as the UK government's Mental Health Recovery Action Plan [31], which aimed to address health inequalities through targeted prevention activities and culturally tailored interventions. However, to sustain such initiatives, funding structures must prioritise long‐term investment in prevention and community‐led solutions, as recently highlighted in the independent review investigation of the national health service in England commissioned by the UK Government [32]. Policymakers should consider adopting evidence‐based, participatory approaches to ensure that resources are allocated effectively and equitably. Important to this process is the underlying adaptation of the PO framework represented in Table 1 that offers principles to support the design of interventions. This helps to make clear links between the design features of an initiative such as Ubuntu House and the underlying principles that support health empowerment.

### 4.3 Limitations and Future Research

While the project yielded valuable findings, several limitations must be acknowledged. Remote methods were used for much of interview stages, limiting the researcher's ability to observe and contextualise participants' experiences within The Centre. This was critical as the research was carried out during a period when pandemic‐era restrictions were in place. Even as restrictions eased, some participants were hesitant to attend in‐person sessions, reducing opportunities for deeper engagement. This reliance on remote methods may have affected the richness of the data and limited the scope of co‐design activities. For example, concerns about safeguarding in a remote context and participants' privacy may have influenced the level of depth the interviews went into. In addition, the researcher relied on the Centre to support them in building rapport with service users, reiterating their cultural competence and shared lived experience. Lastly, the researcher was unable to attend the Centre in‐person while analysing findings from the interviews, which made it challenging to contextualise particiapnts' comments about the design on the space.

The final concept, Ubuntu House, remains a proof of concept and has not yet been tested or implemented. The pandemic, coupled with the project's timeframe and funding constraints, hindered the development of a meaningful implementation strategy. Future research should explore ways to pilot the intervention, such as creating a temporary or pop‐up version of Ubuntu House in communities with a high proportion of Black residents. This would allow for evaluating its feasibility, user engagement, and impact on attitudes toward mental health management. Testing the intervention would also provide opportunities to refine the design and identify best practices for scaling it as a permanent service. The clear link with PO theory and the thinking around empowerment represented in Table 1 would offer a strong basis for informing methods for testing.

Further research is also needed to expand the application of the psychological ownership framework to other marginalised groups and healthcare contexts. Studies focusing on the application of psychological ownership theory through afforded interactions and design process have advanced and improved thinking in how to apply the theory [25, 33, 34, 35, 36, 37]. However, this study highlights the role of cultural identity in ownership, suggesting that interventions must account for the unique experiences and values of different communities. Additionally, the interplay between individual abilities and environmental opportunities warrants deeper exploration to identify how systemic changes can best support ownership. The findings also underscore the importance of co‐creation and user involvement in addressing health inequalities, offering a model for future projects that aim to design culturally competent and sustainable healthcare solutions.

Finally, there are many other areas of empowerment that could be explored using the PO framework as a basis. From data management and new modes of consumption through to wider applications in health, this paper may help to inform other studies that consider how and why one may be empowered to engage with often difficult topics. This offers a rich area of future research within the PO community.

## 5 Conclusion

This project sought to address gaps in mental health support for Black communities by applying the psychological ownership framework as both an analytical lens and a design tool. Through participatory methods, involving members and staff from The Centre, the study explored how individuals develop or fail to develop ownership over their mental health and how these insights could inform the design of culturally tailored interventions. The findings highlighted the systemic challenges faced by Black individuals in engaging with mainstream mental health services, including stigma, cultural insensitivity, and fragmented care. At the same time, they demonstrated the positive impact of person‐centred, culturally grounded recovery models, such as that of The Centre, in fostering empowerment and belonging.

The application of the psychological ownership framework provided a deeper understanding of how motives such as efficacy, self‐identity, and belonging, and routes such as control, intimate knowledge, and self‐investment, shape mental health management. These insights informed the design of Ubuntu House, a proof of concept for a culturally tailored mental health intervention. Ubuntu House integrates the principles of psychological ownership with best practices for community‐based recovery, offering a potential model for addressing health inequalities and empowering Black individuals to take ownership of their mental health.

While the project offers valuable theoretical and practical contributions, it also underscores the need for further research and implementation. Testing Ubuntu House in real‐world settings will be critical to refining the concept and assessing its feasibility and impact. More broadly, the study highlights the importance of meaningful engagement with underserved communities, ensuring that their voices are central to the design and delivery of healthcare solutions. By combining behavioural science theory with participatory methods, this work provides a pathway for creating equitable, user‐centred mental health services that empower individuals and address systemic disparities.

## Author Contributions

**Clarissa Gardner:** conceptualisation, investigation, methodology, project administration, writing – original draft, writing – review and editing, formal analysis. **Weston Baxter:** writing – original draft, writing – review and editing, investigation, conceptualisation, methodology, supervision, formal analysis.

## Ethics Statement

Ethics approval was then obtained from Imperial College Research Ethics Committee (ICREC: 20IC6144).

## Conflicts of Interest

The authors declare no conflicts of interest.

## Data Availability

Data sharing is not applicable to this article as no datasets were generated or analysed during the current study.
